# Shoe-Integrated, Force Sensor Design for Continuous Body Weight Monitoring

**DOI:** 10.3390/s20123339

**Published:** 2020-06-12

**Authors:** Shahzad Muzaffar, Ibrahim (Abe) M. Elfadel

**Affiliations:** 1 Electrical Engineering and Computer Science, Khalifa University, Abu Dhabi, UAE; shahzad.muzaffar@ku.ac.ae; 2 Center for Cyber Physical Systems (C2PS), Khalifa University, Abu Dhabi, UAE

**Keywords:** force sensor design, flexible packaging, shoe-integrated sensing, continuous weight measurement, gait monitoring, Biomedical IoT

## Abstract

Traditional pedobarography methods use direct force sensor placement in the shoe insole to record pressure patterns. One problem with such methods is that they tap only a few points on the flat sole under the foot and, therefore, do not account for the total ground reaction force. As a result, body weight tends to be under-estimated. This disadvantage has made it more difficult for pedobarography to be used to monitor many diseases, especially when their symptoms include body weight changes. In this paper, the problem of pedobarographic body weight measurement is addressed using a novel ergonomic shoe-integrated sensor array architecture based on concentrating the applied force via three-layered structures that we call Sandwiched Sensor Force Consolidators (SSFC). A shoe prototype is designed with the proposed sensors and shown to accurately measure body weight with an achievable relative accuracy greater than 99%, *even* in the presence of motion. The achieved relative accuracy is at least 4X better than the existing state of the art. The SSFC shoe prototype is built using readily available soccer shoes and piezoresistive FlexiForce sensors. To improve the wearability and comfort of the instrumented shoe, a semi-computational sensor design methodology is developed based on an equivalent-area concept that can accurately account for SSFC’s with arbitrary shapes. The search space of the optimal SSFC design is shown to be combinatorial, and a high-performance computing (HPC) framework based on OpenMP parallel programming is proposed to accelerate the design optimization process. An optimal sensor design speedup of up to 22X is shown to be achievable using the HPC implementation.

## 1. Introduction

Pedobarography is the art of measuring and analyzing pressure forces exercised by the foot on the ground during the walk cycle. Wearable pedobarography is a highly promising research area whose results are expected to impact a range of healthcare applications such as gait biometrics, activity monitoring, weight estimation, prosthetics, physical therapy, Parkinson’s disease, neuro-motor disorders, obesity control, and sports medicine. Other engineering areas should profit from wearable pedobarography, including robotics, security, and entertainment.

**Figure 1:**
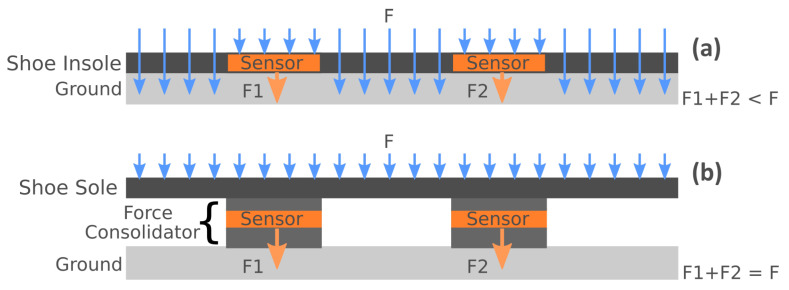
(**a**) Sensors in Shoe Insole (**b**) Sandwiched Sensor Force Consolidators (SSFC).

In the specific context of healthcare applications, it is well known that the short-term change patterns of body weight are correlated with several physical, mental, and emotional health problems. A partial list of medical conditions with such correlations is shown in Table 1. For instance in its self-management plan for heart failure, the American Heart Association considers a sudden weight gain of more than 2–3 lbs in a 24-h period (or 5 lbs in a week) a medical alert in the call-your-physician-or-call-911 category [1]. Such recommendation is supported by medical studies such as [2], which has concluded that for congestive heart failure, “increases in body weight are associated with hospitalization for heart failure and begin at least 1 week before admission. Within the week before hospitalization, the risk of heart failure hospitalization increases in a monotonic fashion with increasing amounts of weight gain. Any weight gain of >2 pounds is associated with an increased risk of heart failure hospitalization. Our results indicate that weight gain is an important risk factor for hospitalization within a relatively short time period. Daily information about patients’ body weight can alert clinicians to patients who are at high risk for hospitalization on the basis of weight gain.” A similar study [3] has concluded that using moving averages for body weight measurement is a better predictor for heart failure hospitalization than spot measurements. It is, therefore, crucial to enable monitoring technologies that can detect such body weight changes early enough so as to prescribe preventive treatments and avoid emergency and intensive care conditions. The continuous monitoring of body weight requires, of course, the continuous measurement of ground reaction forces under the feet. The traditional method to measure such forces is to place force sensors in a shoe insole and collect the sensor data for further analysis. Unfortunately, such methods fail to account for the total applied force due to the force-spreading effect across the shoe insole, which makes it impossible to account for the total reaction force. This is because they tap only a few points under the flat insole. This force-spreading effect is illustrated in Figure 1a.

Researchers have used optimization to suitably place shoe-integrated FlexiForce pressure sensors under the feet. However, such optimizations cannot be reliably generalized due to person-to-person gait and foot variabilities. A comparative study of footwear-based wearable systems [4] lists several other approaches where shoe-integrated sensors are used for gait analysis and possibly also for weight estimation. However, these approaches, while innovative, do not accurately collect the total ground reaction force (GRF), nor do they account for the various sources of measurement noise and error. One possible approach to improve the accuracy of the estimated subject weight is to eliminate the direct contact of shoe-mounted sensors with the ground. Such an approach is adopted in several studies, but unfortunately, their actual implementations result in unacceptably large form factors that make them unsuitable for a flexible, comfortably wearable, low-cost, and low-power instrumented shoe design. Recently published work on shoe-wearable weight estimation systems illustrate the steady progress that the field has made and the challenges that remain to be addressed. One major challenge is the large weight estimation error that is due, in large part, to inaccuracies in total GRF measurement. One potential solution is the full sensory shoe insole from Tekscan [5] which, while it covers most of the foot pressure area with a dense array of pressure sensors, it still cannot be used for accurate weight estimation. Indeed the comparative study conducted in [6] indicates a significant difference between the absolute ground reaction force measured using a Tekscan insole and that of a force plate. While specialized calibrations may be used to correct some of these differences, their usage is restricted to laboratory research. The calibration techniques, the bulky wiring harness to interface with the sensor array, and the costly insole material are all limiting factors to their widespread wearability and adoption in the cost-sensitive healthcare market. More details about the state-of-the-art in shoe-based force data collection and analysis will be given in the next section.

The main purpose of this paper is to address the above challenges in wearable body weight estimation using a novel approach to shoe-integrated sensor design and characterization. The main sensor design innovation is the integration of force sensors in three-layered structures that act as force consolidators and channel the total applied force to a set of critically placed points under the foot. The second design innovation is a semi-computational methodology for the fast extraction of transduction characteristics of the sensing layer in the multi-layer structure. Driven by ergonomic and comfort considerations, such semi-computational approach helps accelerate the design iterations of multi-layer sensing structures of arbitrary shape, while mitigating the inefficiencies of manual designs based on custom fabrication or automated designs based on full 3D finite-element-method (FEM) analysis of the shoe-integrated sensor.

The main contributions of this paper are as follows:

1. *Sensor Design:* A novel method for designing the force sensors is proposed. A layered structure called *Sandwiched Sensor Force Consolidators (SSFC)* is used under the shoe sole to concentrate and channel the applied forces. Each SSFC is a three-layer structure made of a top and bottom capping layers, and a middle sensory layer, as shown in Figure 1b. The proposed method can be used to customize the SSFC shape in support of an ergonomic shoe design that is comfortable to wear.
2. *Feasibility Analysis and Proof-of-Concept:* A fully instrumented proof-of-concept shoe prototype has been built and tested on a human subject. It has been shown to achieve a weight estimation accuracy greater than 99%, which is at least 4X better than the state-of-the-art [7,8,9].
3. *Ergonomic Shoe Design and Custom Sensor Characterization:* To improve the wearability comfort of the proof-of-concept prototype, the paper introduces a streamlined, efficient, equivalent-area methodology for the extraction of the transduction characteristics of SSFC sensor structures of arbitrary shapes. The methodology is fully self-contained and does not require external or vendor 3D FEM codes for shape-from-force analysis.
4. *High-Performance Processing:* To speed up sensor characterization and the design optimization loop of the semi-computational methodology, a parallel programming interface has been implemented and tested using C and OpenMP. It has been shown to reduce the shoe design optimization runtime from few days to few hours.

The remainder of this paper is organized as follows. Section 2 surveys the prior art in footwear sensors. Section 3 describes the proposed sensor architecture. With the goal of achieving an ergonomic, comfortable shoe design, Section 4 presents an efficient, semi-computational characterization framework for the design of sensor arrays having sensors of arbitrary shapes. Section 5 provides the implementation details of both the instrumented shoe prototype and the sensor design methodology. In Section 6, various experimental and characterization results are provided to illustrate the overall soundness of the proposed sensing and sensor design concepts. The conclusions are given in Section 7.

## 2. Review of Prior Art

The literature on shoe-integrated sensors is rather large and goes back to the seminal work of [10]. Given the paper’s focus on continuous bodyweight monitoring and the dependence of such monitoring on the measurement of ground reaction forces (GRFs), our review of the prior art is restricted to the more recent references that have addressed the use of shoe-integrated pressure sensors for measuring GRFs.

Broadly speaking, the prior art in this area can be categorized into two categories. The first comprises the sensing systems that end up collecting only a portion of a GRF while the second comprises those that collect the total GRF. To the first category belong the applications that target the observation of relative foot pressure patterns without much dependence on the exact magnitude of the ground reaction force. Research in gait analysis and recognition as described in [11,12,13,14,15], belongs to this category. Also within this category are the shoe-integrated sensing systems where the pressure and force sensors are assisted with range [16] or inertial [17] sensors. In robotics, pressure sensors along with motion sensors are used in the motion monitoring and control of biped robots [18]. In healthcare, a similar combination of sensors is used in the commercial smart shoe from [19,20], which is designed to provide real-time gait analysis. Other commercially available instrumented shoes include [21] for athletic performance monitoring and training, and [22,23] for health analytics. Although the commercial vendors have not disclosed the technology of their shoe-integrated pressure sensors, all the above sensory systems use, to the best of our knowledge, the Force Sensitive Resistor (FSR) sensor, which is based on the piezoresistive transduction. Besides the type of sensor used, another common feature amongst the above systems is the use of the shoe insole as the location of pressure sensor integration. The only exception is [21], which uses a smart sock. Given their partial GRF collection, these shoe-integrated force sensing systems are more suitable for gait and activity monitoring rather than continuous body weight measurement.

The second category of the prior art, where the measurement targets the *total* GRF, has more variety in its transduction modalities. This is because the main goal of shoe-integrated, total GRF measurement has been to develop an ambulatory equivalent of the static force plate system, which is considered the golden standard in bio-mechanical and sports medicine research [13,24]. As a result, more sensing mechanisms have been explored, including mechanical, optical, tactile, and piezoelectric. An early example of a mechanical GRF measurement is [25] in which the use of insole-embedded air pressure sensors has been proposed for gait monitoring. The main advantage of the air pressure sensors is their high sensitivity to pressure changes across the contact surface between shoe and ground, which improves the quality of the total GRF measurement. On the other hand, the air pressure measurement system is bulky, requires inordinate amounts of power, and therefore is not easily wearable. A more recent example that also uses air pressure sensors for GRF measurement is [26], where the target application is human activity classification. In addition to air pressure sensors, this system uses four inertial motion units to assist with activity identification by measuring both GRF and knee joint angles. In terms of GRF measurement, the advantages and disadvantages of this sensor system are similar to those of [25]. The optical sensing modality of GRF is best illustrated in the recent work described in [7,27], where a multi-axis fiber-optic force sensor is proposed for the concurrent measurement of normal and shear forces with film-based pressure sensors used to generate moment measurements. In [7], this opto-mechanical sensor system is applied to measure the total GRF with an accuracy above 90% in comparison with standard force plate measurements. Unfortunately, this highly accurate GRF measurement sensor is of limited wearability and comfort because of the need to sandwich it between two metal plates placed under the shoe. Furthermore, the drive and readout circuitry is not easy to integrate, especially that several power-hungry components are needed for the operation of the sensor. Another example of the use of optical sensors for total GRF measurement is [28], where five optical tri-axial force sensors are encapsulated on the bottom of the shoe sole for the monitoring of extrinsic gait variability. This shoe-integrated system has the same drawbacks as [7,27]. The tactile sensing modality has been used in [24], where a dense array of small, low-cost, 3-D tactile sensor cells are placed under the entirety of the shoe sole for measuring the total GRF. The drawback of this measurement system is the bulkiness of the wiring system and the complex readout circuit that must handle a multitude of sensors in real-time. It is interesting to note that all the shoe-integrated platforms for total GRF measurements that use the mechanical, optical, or tactile transduction mechanisms have their sensors placed on the external frame of the shoe rather than in the insole.

A counterpart of [24] for the insole is the commercially available [5], which uses a dense array of FSRs in an interesting attempt to capture the total GRF. Due to the bulkiness of the wiring harness, such insole is mainly used in laboratory research and is not suitable for wearable applications. In terms of accurate weight estimation, a benchmarking study [6] has shown that when the force plate measurement is used as the golden standard, the density of the insole-integrated array of force sensors is not in itself sufficient to capture the total GRF accurately. Other total GRF studies such as [29,30,31] that have used [5] as a golden standard may, therefore, need to be reassessed.

Bodyweight measurement using GRF has been directly addressed in [8,9] where FSR sensors have been placed into the insole with the explicit goal of measuring body weight. However, due to partial GRF collection, both methods result in significant weight estimation errors, especially when the subject is moving. One promising approach is that of [32] where a nanocomposite piezo-responsive material is proposed as a sensing medium integrated under the shoe insole for 3D GRF measurement during walking. While the accuracy results were comparable to other total GRF measurement systems such as [28], system-level issues such as sensor synchronization were challenging to overcome. All the reviewed literature has been summarized in Table 2, where two additional columns related to custom sensor design and technology readiness have been added for the sake of completeness. The work described in this paper has been contrasted with all the prior art in illustration of the fact that our proposed shoe-integrated bodyweight measurement system is the first to use FSRs on the external frame of the shoe for medical body weight monitoring. Our approach is unique in that a custom sensor design procedure is introduced to increase shoe comfort and improve its wearability. All these aspects will be explained next.

## 3. Novel Force Sensor Structure

**Figure 2:**
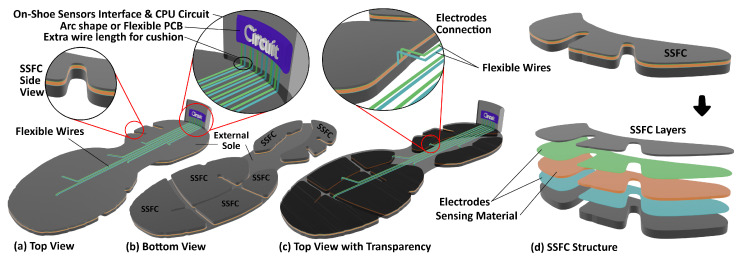
Custom Shoe Designs with 6 SSFCs.

We propose a method for shoe-integrated sensor array design to mitigate the “spreading-out” effect of the total GRF. The main idea is to concentrate the applied forces on a set of critical points placed under the foot. To do so, we build *Sandwiched Sensor Force Consolidators (SSFC)* under the shoe sole, as shown in Figure 1b. The number and shapes of the SSFCs can be customized, which helps in optimizing the shoe design for both comfort and force sensing accuracy. To illustrate the fact that the SSFCs based shoe can be as comfortable as a commercially available design, we have used the sole style of Mizuno’s “Wave-Rider 20 Osaka” and created the 3D model shown in Figure 2. The SFFCs will be integrated into this design style as will be presented in the subsequent subsections.

### 3.1. SSFC Structure

The SSFCs are attached to the bottom side of the shoe sole, as shown in Figure 2a–c. Note that with this configuration, the shoe sole is not in direct contact with the ground. Only the SSFCs make direct contact with the ground. Each SSFC is composed of three core layers with the top and bottom layers sandwiching the sensing layer, as shown in Figure 2d. The top and bottom layers can be manufactured with the same material as the shoe sole. The sandwiched sensor layer can be made of a transduction material (i.e., capacitive, resistive, piezoelectric, etc.), that is used to convert the applied force or stress to a measurable electrical quantity. The sensing layer has top and bottom electrodes that act as ports for electrical measurements. It is reemphasized that the sensing layer is encapsulated and makes no direct contact with either the ground or shoe sole.

### 3.2. On-Shoe Circuitry and Connectivity

**Figure 3:**
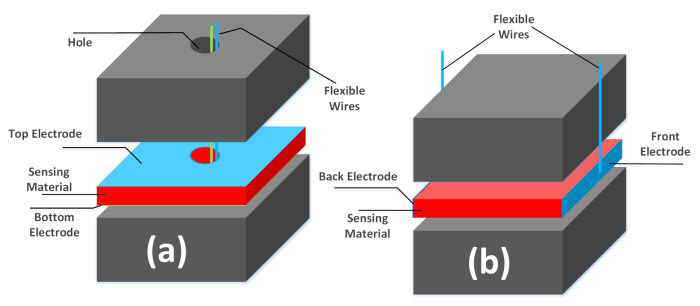
A Few More Embodiments [33,34]: (**a**) Using a Hole in the Top and Sensory Layers (**b**) Using Sides of the Sensing Material.

To facilitate contact with electrodes, the shoe insole is used to pass flexible wires with sideway connection with the electrodes, as shown in Figure 2a,c. The sensor interface and processing circuitry are placed in the heel counter of the shoe. The sideway connection is one possible embodiment for contacting electrodes using the thickness of the sensing material. Another embodiment is to pierce a hole in the top and sensory layers to pass the flexible wires, as shown in Figure 3a. The width or length of the sensing material can also be used in yet another connectivity embodiment, as shown in Figure 3b, in which the electrodes are placed at the two sides of the sensing material without piercing any hole in the consolidator layers. In all cases, the wires pass either through the shoe insole or through the sides to connect the sandwiched sensors with the processing circuitry.

### 3.3. Array of SSFCs

As will be explained shortly, we have determined that 6–8 consolidators, properly located in the sole, are sufficient to collect the total GRF. This is the design guideline we have implemented in our prototype and used in all our experiments. It should, however, be understood that under one shoe, there can be as many SSFCs as needed. A larger number of SSFCs can be used to fabricate a grid of sensory consolidators that help to capture the two-dimensional distribution of the local GRFs. Such distributions can be analyzed in a plethora of applications other than body weight measurement and walk cycle analysis. These applications include fall detection, body balance, and gait analysis. In our prototype, each SSFC sensor has an applied-force rating in the range of 220–445 Newton, but of course, other ratings are possible as may be the case for the physical therapy of a toddler or the monitoring of post-stroke body balance of an obese patient.

### 3.4. Proof-of-Concept Prototype

**Figure 4:**
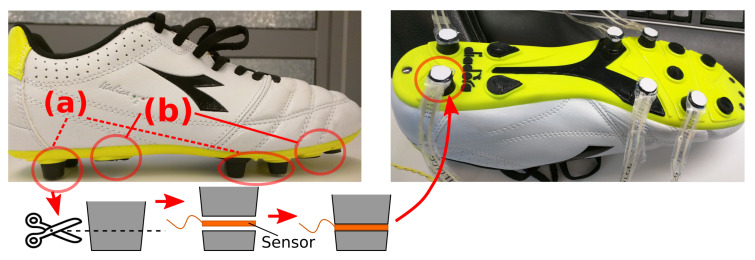
Proof-of-concept Prototype Shoe Based on SSFCs (**a**) Cleats (**b**) Removed Extra Cleats.

In this subsection, we briefly highlight the implementation of a proof-of-concept prototype shoe where we have used the cleat-like structure of soccer shoes and the piezoresistive force sensors, FlexiForce, to create SSFCs according to the concept proposed in Section 3. The process is illustrated in Figure 4. We have selected the soccer shoes, and the FlexiForce sensors because they are available off the shelf and can be readily adapted to develop the feasibility data acquisition prototype, which will show the validity of our sensor design concept and method. The experiments have been performed to verify the effectiveness of the proposed design in collecting the total GRF. The prototype implementation and experimental details are presented in Section 5.

## 4. Improving Comfort and Wearability

**Figure 5:**
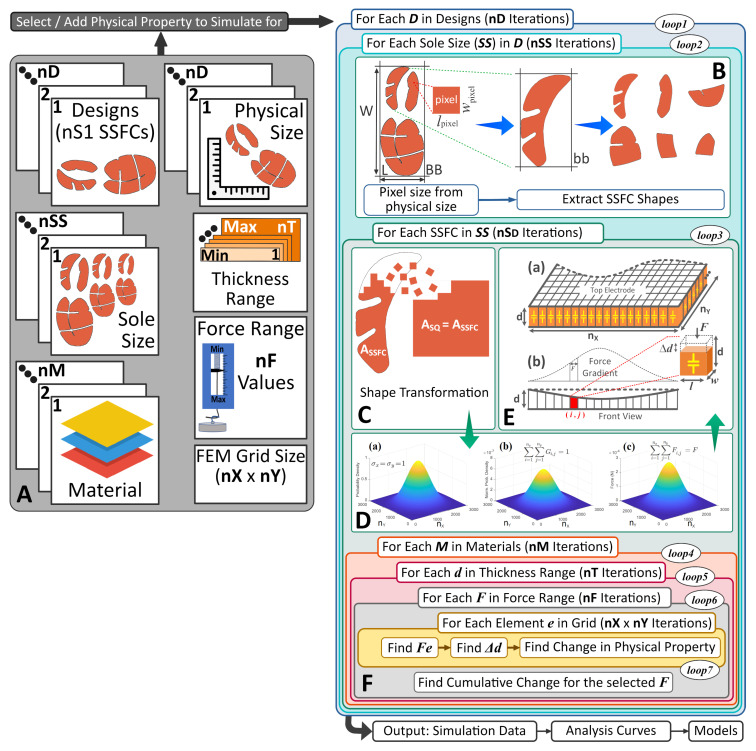
Process Flow Diagram: **A**—System Input, **B**—SSFCs Preparation, **C**—Shape Transformation to a Regular Shape, **D**—(**a**) Gradient of Normal Distribution (**b**) Normalized Gradient (**c**) Mapped Force Gradient for F=450N, **E**—(**a**) Grid of *N* Capacitive Elements (**b**) Thickness of Elements under Force Gradient., **F**—Cumulative Change for a Selected Force.

The soccer-like shoe used in the prototype of the previous section is of course not suitable for comfortable daily wear, especially in the context of continuous monitoring of a patient’s weight and gait. In this section, we show that the SSFCs don’t have to be embedded in cleats but that a more ergonomic and comfortable sensor array design is feasible. We do so by introducing a semi-computational approach for designing SFFCs of arbitrary shapes. The challenge is to show that the transduction characteristics of such custom SFFCs will satisfy the requirements of an accurate ground-force reaction sensor array. As mentioned, SSFCs are necessary to address measurement errors due to the spreading-out effect of the total ground reaction force. However there are other sources of measurement errors. One such source is the potential inaccuracy in the transduction coefficient of the SSFC sensing layer itself. This transduction coefficient depends on the shape and size of the consolidator, the materials used therein, and their layer thicknesses. The transduction coefficient may be determined either empirically or computationally. The empirical method is based on the direct measurement of a fully fabricated consolidator sample. Such a method is tedious, wasteful of materials, and is not compatible with design iteration or optimization. The computational method typically uses 3D FEM codes which require the meshing and analysis of complex 3D shapes such as the ones shown in Figure 2. In a design optimization loop where the goal is to optimally customize a shoe over multiple designs, sizes, SSFCs per sole, sensing materials, and layer thicknesses, thousands of design/modeling iterations are needed. For instance, in the case of 4 designs, 3 sizes, 6 SSFCs for each design, 5 materials, and 10 thicknesses for each material, the combinatorial search space has 3600 possible designs. In such cases, the evaluation of the mechanical and transduction properties of the design at each iteration using the FEM method is time-consuming, and its inclusion in the design iteration and optimization loop is not practical. We, therefore, introduce a semi-computational approach based on the equivalent square area of the consolidator shape where few modeling approximations are applied to simplify and speed up the numerical validation process. As will be shown, the semi-computational approach is self-contained and very amenable to parallelization, which makes it computationally efficient and therefore practical to include in a sensor optimization loop. The proposed framework is specifically meant to accelerate an iterative design process focused on achieving comfort and wearability. The semi-computational approach, whose flow is shown in Figure 5, is based on three main modeling approximations:

1. Sensor shape pixelization and mapping onto a square of equal area.
2. Z-direction discretization of the sensing material thickness.
3. Rotationally symmetric force distribution over the equivalent square area.

The end goal of the SSFC design process is to conjure up, in the shortest possible time, an ergonomic, comfortable shoe design capable of accurate body weight measurement. An important outcome of the SFFC design process is the transduction model of the sensor array that enables the instrumented shoe to determine in real-time the ground reaction force. The steps involved in the design and characterization process are explained in the following subsections.

### 4.1. Design Specifications

The specifications of the SFCC shoe include the number of shoe sole designs (*nD*), the number of SSFCs per design (*nS_D_*), the number of shoe sizes (*nSS*), the physical size of each design ($W_{phy}\times L_{phy}$), the number of materials (*nM*), the sensing layer thickness range (*nT*), the expected force range (*nF*), and the FEM grid size ($N=n_{X}\times n_{Y}$). These specifications are illustrated in Figure 5A. Besides design specifications, inputs into the design methodology include *nD* high-resolution images that are either camera-captured or computer-generated. Additionally, the required properties of all the *nM* materials such as the resistivity, Young’s modulus, piezoelectric coefficients, and relative permittivity constant, must be provided. The methodology implementation allows the selection of any physical property to monitor across all the candidate materials and shape designs. The design search space at this step is comprised of a nested combination of $nD\times nSS\times nS_{D}\times nM\times nT\times nF\times n_{X}\times n_{Y}$ cases. The computational work performed for each sole size in each shoe design is described next.

### 4.2. Image Processing and Equivalent Area

A high-resolution image of a shoe sole is captured or generated, and the dimensions of the sole bounding box (BB) are extracted from the image using the start and end pixels of the columns ($C_{Start},C_{End}$) and the rows ($R_{Start},R_{End}$), Figure 5B. The bounding box length, *L*, and width, *W*, are given by

(1) $$L=C_{End}-C_{Start}+1,\;W=R_{End}-R_{Start}+1$$

for a total area of L×W pixels. The pixelization of the bounding box is based on pixels whose length, $l_{pixel}$, and width, $w_{pixel}$, are given by

(2) $$l_{pixel}=\frac{L_{phy}}{L},\;w_{pixel}=\frac{W_{phy}}{W}$$

This pixelization is needed to map the shoe sole *image contour* to that of the shoe itself. Next, the bounding box (bb) of the individual SSFC and its dimensions are extracted as shown in Figure 5B.

Each SSFC shape is then transformed into a square (other regular shapes are also possible) whose area $A_{SQ}$ is the same as that of the SSFC shape (i.e., $A_{SQ}=A_{SSFC}$) with a side length $L_{SQ}=W_{SQ}=\sqrt{A_{SSFC}}$, Figure 5C. $A_{SSFC}$ is determined by counting the number of pixels covered by the SSFC shape and using the simple formula $A_{SSFC}=PixelCount\times l_{pixel}\times w_{pixel}$. The advantages of using the equivalent-area transformation include:

1. *Computational Efficiency:* As will be explained shortly, the SSFC is meshed into small elements, and the square transformation eliminates the need to access elements outside the sensing area. For example, the shape in Figure 5C has more than 50% area that needs no processing. Even if the elements in this area are marked as not-to-process, at each iteration, at least one memory access is needed to check the element validity. There could be millions of such elements during the full simulation process, which will waste a substantial amount of processing time.
2. *Algorithmic Simplification:* The quantity of interest is the cumulative change in the physical property of an SSFC rather than the change gradient across its surface. Therefore, preserving the SSFC geometry is unnecessary. This helps in simplifying the computation of additional effects such as the fringe effects in capacitive sensors. It is well known that it is quite complex to account for the fringe capacitive effects of an irregular shape, while a regular one allows the use of existing true-and-tried formulas.
3. *Realistic SSFC Simulation:* The shape transformation further enables the use of force distribution models that result in realistic vertical displacement maps and, therefore, realistic SSFC transduction characteristics.
4. *Reduction of Approximation Errors:* Finally, it helps in reducing the approximation errors inherent in the meshing of a complex shape and the discretization of its boundary.

### 4.3. Force Distribution Model

The force distribution model is meant to mimic the forces that are applied across the SSFC during a given phase of the walk cycle as, for instance, during heal impact. The model is a two-dimensional distribution whose definition is under the control of the designer. It is defined over the equivalent square area as introduced in the previous paragraph. As shown in Figure 5(E-a), the equivalent square is gridded using $N=n_{X}\times n_{Y}$ elements, whose number is also under the control of the designer and reflects the spatial accuracy with which the SSFC transduction characteristics are calculated. The length and width of each element *e* in the grid are given as:

(3) $$l=\frac{L_{SQ}}{n_{X}},\;w=\frac{W_{SQ}}{n_{Y}}$$

The force distribution function itself may be either deterministic or stochastic. For illustration purposes, a rotationally symmetric normal distribution function is used in this paper as shown in Figure 5(D-a). The normal distribution is centered at the center of the equivalent square and has a standard deviation of one grid element ($\sigma_{x}=\sigma_{y}=1$). The discretized values, $G_{i,j}$, of this distribution over the $n_{X}\times n_{Y}$ grid are normalized to sum up to 1 (i.e., $\sum_{i=1}^{n_{X}}\sum_{j=1}^{n_{Y}}G_{i,j}=1$). When a force *F* is applied, it will be distributed across the equivalent square according to $F_{i,j}=F\times G_{i,j}$. The force mapping is illustrated in Figure 5(D-b,D-c). It is this force value, $F_{i,j}$ at the (i,j) element that will be used to compute the displacement and the electrical transduction variable at the (i,j) location of the equivalent square, as shown in Figure 5E. For example, if we are simulating SSFC for the capacitive change, the transformed shape would be divided into a grid of *N* mini capacitors between the top and bottom electrodes of the SSFC, as shown in Figure 5(E-a). The application of the force gradient would result in variable thicknesses across the grid, as shown in Figure 5(E-a).

### 4.4. Transduction Characteristics

The above process is repeated for each SSFC in a given sole size and shoe design for $nD\times nSS\times nS_{D}$ iterations (i.e., loop1×loop2×loop3) in Figure 5. In each *loop3* iteration (i.e., for each SSFC), the computation of transduction characteristics is based on a set of four nested iterations. The outermost iteration, *loop4*, is over the materials, *M*, provided by the designer. For each *M*, the second iteration, *loop5*, is over the thickness of the material, *d*, for a range also set by the designer ($d_{min}\leq d\leq d_{max}$ with a step size δd). For each *d* in the thickness range, the third iteration, *loop6*, runs over the applied force *F* for a user-defined force range (i.e., $F_{min}\leq F\leq F_{max}$ with a step size δF). Finally for each force, the innermost iteration, *loop7*, goes over all the *N* elements of the equivalent square grid. Denoting by $n_{F}$ and $n_{T}$ the respective numbers of iterations in the force and thickness ranges, the total number of iterations executed to compute the full transduction characteristics is $n_{F}\times n_{T}\times N$ iterations.

The change in thickness, $\Delta d_{i,j}$, at a grid element (i,j) is determined as follows [35]

(4) $$\Delta d_{i,j}=\frac{F_{i,j}\times d}{Y\times w\times l}$$

where *Y* is the Young’s modulus of the sensing material. The change in the electrical transduction variable is determined using one of the equations in Table 3 according to the selected transduction mechanism. At the end of the innermost *N* iterations, all the elements have been processed for the force and thickness values given by the outer iterations. All the changes are then combined to generate an aggregate change in transduction variable of the SSFC, as shown in Figure 5F. The aggregation method depends on the placement of electrodes. As for the illustration purpose we are simulating for capacitive change and have placed electrodes at the top and bottom of SSFC, all the *N* capacitances in the grid are in parallel and are added to find the aggregate change in SSFC capacitance. The total change in the capacitance (transduction variable) can be measured and converted to a suitable electrical change using appropriate electronic circuitries. As will be shown in Section 6, at the end of the outermost *loop1*, the computation results in $nD\times nSS\times nS_{D}\times nM\times nT$ force transduction functions. Each function describes the relationship between the applied force range and the aggregate change in the transduction variable for a given thickness of the sensing material. It is important to note that the formulas of Table 3 are based on simplifying assumptions that may not hold for very small sensing elements. For instance, in the case of capacitive sensing, when the dielectric thickness is of the order of magnitude of the dimensions of the sensing area, the use of the parallel-plate capacitance of Table 3 may not be justified, and as a result edge effects will have to be accounted for in the capacitance calculation. This aspect will be amply illustrated in the next section.

## 5. Implementations

In this section, we will present the implementation of both the feasibility prototype to validate the proposed sensor design approach, and the computational framework to accelerate the process to design ergonomic shoes based on our sensor approach.

### 5.1. Feasibility Prototype

**Figure 6:**
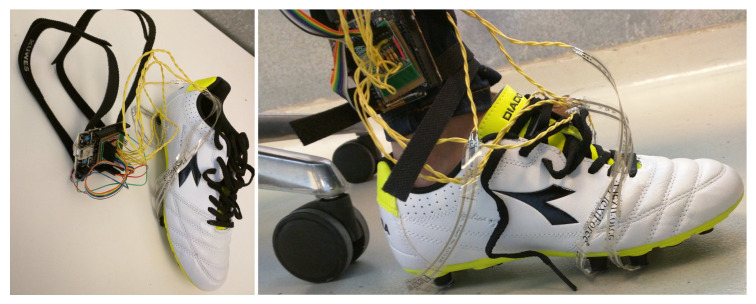
Prototype Data Acquisition System.

**Figure 7:**
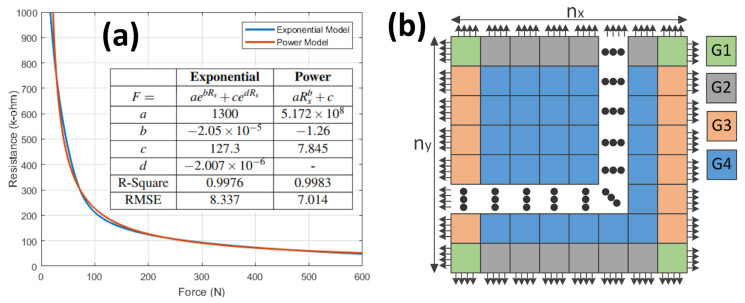
(**a**) FlexiForce Sensor Model Plot (**b**) Groups of Elements.

The prototype data acquisition system, shown in Figure 6, comprises several hardware and software components. The sensor array subsystem uses FlexiForce force sensors which measure the consolidated forces under the foot via the impedance changes due to the stresses applied to the sensing material. Six FlexiForce force sensors are integrated into the spikes of a soccer shoe. The sensors and the spikes together act as sandwiched sensor force consolidators (SSFC) and concentrate the applied forces at selected points under the shoe. The shoe-mounted FlexiForce force sensors change their resistances as per the applied forces. Resistance changes are then converted to voltage using voltage dividers. These voltages are next fed as analog inputs to a six-channel ADC that quantizes them to a discrete number of voltage levels. These levels are then sent to the force calculator where all the six voltages of the six SSFC’s are used to find the sensor resistances. Subsequently, the transduction characteristics are applied to find the corresponding forces. In the present prototype, the transduction characteristic is computed using the manufacturer’s resistance vs. force plot for the FlexiForce sensor. Curve tracing is used to extract the data points from the manufacturer’s plot. The generated data points are then used to model the sensor behavior via curve fitting using exponential and polynomial functions. The resulting exponential and polynomial models are shown in Figure 7a. The six measurements determined using the model block are summed up to find the total GRF. The processing module within the data acquisition hardware is used to implement all the processing steps mentioned above. The force measurement algorithm continuously samples the sensor-induced electrical changes, processes the samples to estimate the GRF, and transmits the raw samples and the force estimate to a computer via UART for further analysis.

### 5.2. Computational Framework—Accelerating the Ergonomic Design

In this section, the semi-computational methodology based on the area-equivalent approach is implemented using a high-performance computing (HPC) framework. The implementation comprises two components:

1. A computational efficiency component to illustrate the significant reduction in processing time for ergonomic shoe design process.
2. A characterization component to illustrate the accuracy of the sensor characterization technique.

As highlighted earlier in Section 4, the optimization of a single shoe design may take thousands of design iterations. To speed up the computation of the transduction characteristics, a C/OpenMP parallel programming interface has been used [36]. The computation flow is divided into three parallel tasks: shape transformation (ST), force distribution (FD), and simulation (SIM). Each task is divided into a grid of threads of dimension $n_{th}\times n_{C}$ where $n_{C}$ denotes the number of cores, and $n_{th}$ denotes the number of threads assigned to each core. The designer sets $n_{C}$ before the start of the computation. The number of threads, $n_{th}$, for each of the three parallel tasks is calculated as follows:

(5) $$n_{th}=\left\{\begin{array}{cc} \frac{Image\;Resolution}{n_{C}}, & \mathrm{if}\;ST\\ \frac{N}{n_{C}}, & \mathrm{if}\;FD\\ \frac{n_{F}\times n_{T}\times N}{n_{C}}, & \mathrm{if}\;SIM \end{array}\right.$$

The thread work for the force distribution task is the least complex while the simulation thread work is the most complex. The runtime of one SSFC is mainly dominated by the simulation task.

To validate the area-based computation of transduction characteristics, we have selected a circular capacitive sensor similar to the one from SingleTact [37]. The main reason for selecting capacitive sensing is to illustrate both the area-equivalent technique and the straightforward handling of the fringe effects that results from mapping the SSFC onto a regular shape. The sensor is transformed into a square shape, and a grid of small elements is created. We have considered the edge elements of the grid to include their edge-effect in the capacitance calculations as these are the only elements which have open sides and participate in fringing. The mesh elements are therefore grouped into four groups $G_{1},G_{2},G_{3},$ and $G_{4}$, as shown in Figure 7b. The effective length and width of each element due to the edge-effect can be determined as follows

(6) $$l_{i,j}^{eff},w_{i,j}^{eff}=\left\{\begin{array}{cc} l_{i,j}+\frac{\Delta L}{2}\;,\;w_{i,j}+\frac{\Delta W}{2} & \mathrm{if}\;(i,j)\in G1\\ l_{i,j}+\frac{\Delta L}{2}\;,\;w_{i,j} & \mathrm{if}\;(i,j)\in G2\\ l_{i,j}\;\;\;,\;w_{i,j}+\frac{\Delta W}{2} & \mathrm{if}\;(i,j)\in G3\\ l_{i,j}\;\;\;,\;w_{i,j} & \mathrm{if}\;(i,j)\in G4 \end{array}\right.$$

where ΔL represents the overall increase in effective length and width of the square due to the edge-effect and can be determined using Palmer’s classical formula for the capacitance of a rectangular parallel-plate geometry [38].

(7) $$\Delta L=\frac{d}{\pi }\left(1+\ln\left(\frac{2\pi L}{d}\right)\right)=\Delta W$$

$l_{i,j}^{eff}$ and $w_{i,j}^{eff}$ are then used in the expression of Table 3 to find the effective capacitance, $C_{i,j}^{eff}$. Recent examples of the use of Palmer’s formula, published in 1937, include [39,40].

The change in geometric shape during the shape transformation process does also affect the fringe capacitance, which must be adjusted accordingly. This is because while the area remains the same, the perimeter changes. To account for such change, the fringe capacitance ($C_{f}$) is scaled by a shape factor (β) that is the ratio of the original shape perimeter, $P_{O}$, to the transformed shape perimeter, $P_{T}$, i.e., $\beta =P_{O}/P_{T}$. The final expression of the capacitance that takes into account perimeter change is

(8) $$C=C_{S}+\beta \left(C_{eff}-C_{S}\right)$$

where $C_{S}$ is the capacitance without considering the edge-effect.

## 6. Experimental Results and Analysis

We have performed several experiments to validate the proposed sensor design approach and the design process acceleration platform. These are given in the following subsections.

### 6.1. Experiment 1

**Figure 8:**
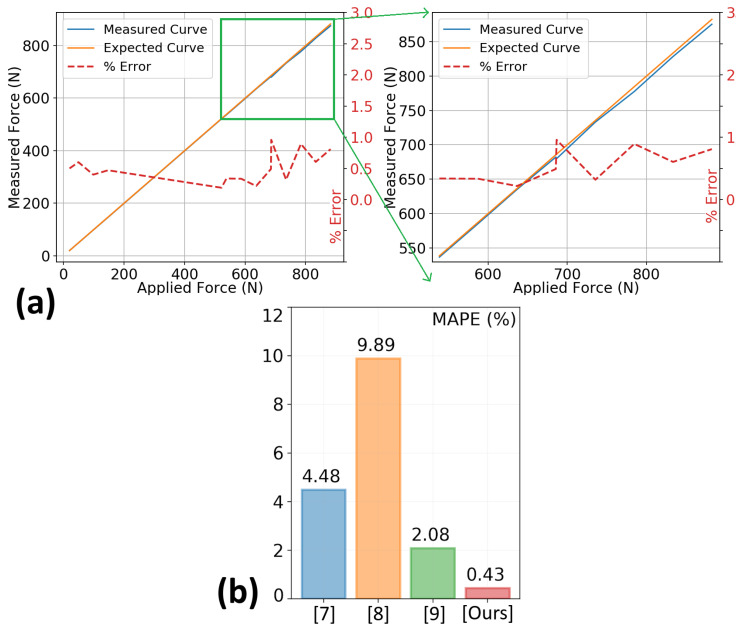
(**a**) Prototype Experimental Results (**b**) Accuracy Comparison: Wearable Weight Estimation Systems.

The wearable data acquisition prototype, presented in the previous section, is used to record the ground reaction forces under two tests. The first is a static test that consists of placing known static weights over the shoes. The second test is with a walking human subject (The subject gave informed consent and the study was reviewed and approved by the Human Subjects Research Ethics Committee (HSREC) at the Masdar Institute, now part of Khalifa University, Abu Dhabi, UAE, under Protocol HSREC#2013-16.), wearing the instrumented shoes. Under the second test, the measurements are taken whether the subject is walking or standing. The subject is 5${}^{\prime }$6${}^{\prime \prime }$ in height and weighs 54.9 Kg with a shoe size of 7 (US). In the experiments, the weight of the subject is incrementally increased using a bag-pack that is loaded with static weights up to a total of 85 Kg. The continuous force measurements are recorded while the samples are processed in real-time to analyze the walk-cycle waveform. The analysis of the measured waveforms shows that the problem of partial force collection that results from the force spreading is eliminated, and the measurements are very close to the applied GRFs. The experiments are repeated 50 times, with each experiment lasting from one to two minutes. The measurement results from one such experiment are shown in Figure 8a. In all the trials, the achieved weight estimation accuracy is higher than 99%. Using the Mean Absolute Percentage Error (MAPE) as a comparison criterion, this achieved accuracy is 10.4, 23, and 4.8 times better than the accuracies reported in [7,8,9], respectively. The primary sources of these small errors include the sensor noise, the accuracy margin of the empirical model, and the imprecise sensor placement within the spikes. If these noise sources are reduced, the errors will decrease. The accuracy of the SSFC prototype is further compared with weight estimation methods have recently been published. The comparisons are given in Figure 8b and show that the SSFC performs significantly better than the state-of-the-art.

### 6.2. Experiment 2

**Figure 9:**
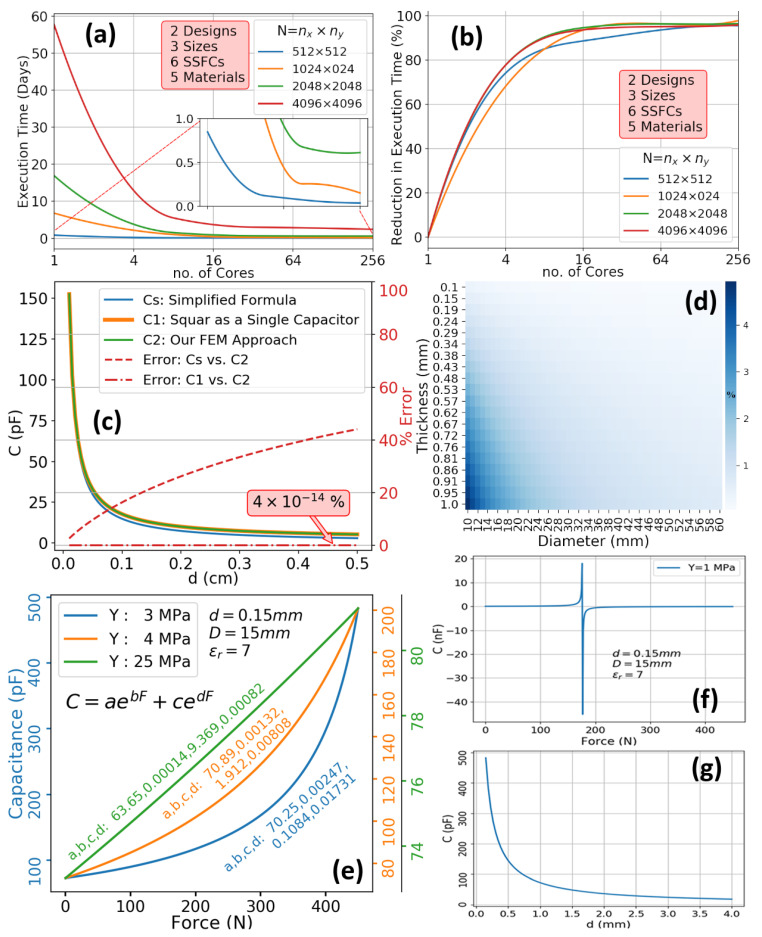
Testing and HPC Simulation Results. (**a**) Execution Time (**b**) Speedup (**c**) Error Comparison (**d**) Error Due to Geometry Change (**e**) Calibration Curves (**f**) Sensing Material Suitability Test (**g**) Capacitance vs. Material Thickness

The functionality of the OpenMP parallel implementation of sensor characterization is verified both on a personal computer with four cores and at Khalifa University’s HPC cluster. A range of data sets of various sizes is used in the numerical experiments to compute the SSFC transduction characteristics and their computation times. Several runs are conducted while varying the number of processor cores. The number of processor cores and data sizes are shown in Figure 9a. As shown in Figure 9a, the characterization using one core (serial execution) may take more than 55 days for a design case with five materials, two shoe designs, three sizes, and six SSFCs in each design. Over an HPC cluster (parallel execution with OpenMP), the execution time for the same case is reduced to less than 2 and a half days, a speedup of 22X. The OpenMP implementation of the semi-computational methodology is dynamic in that it optimizes the parallelism automatically as per the given number of cluster cores to achieve maximum performance gains. The achieved reduction in execution time remains more than 75% in all cases and reaches a maximum of about 98%, as shown in Figure 9b.

An alternative compute infrastructure to the HPC cluster is a graphics processing unit (GPU) running on a personal computer with minor GPU-specific changes in the OpenMP directives of the code.

### 6.3. Experiment 3

To validate our sensor characterization method, we have selected a circular SSFC of diameter *D* = 15 mm with a capacitive sensor of dielectric thickness *d* = 0.15 mm, and dielectric constant $\epsilon_{r}=7$. The dimensions are equivalent to a commercially available sensor from SingleTact [37]. In one experiment, a sensor thickness sweep has been conducted to determine sensor capacitance for three different cases: (1) full square using the parallel-plate formula without edge effects, (2) full square using Palmer’s formula, and (3) grid of small elements using the approach of Section 4.4. The results are compared in Figure 9c. As the thickness is increased, the error with the simplified formula increases. On the other hand, when the grid-based algorithm of Section 4.4 and the Palmer’s formula are compared, their relative difference is $4^{-14}\%$, almost zero. Another parameter sweep has been conducted using both the thickness and the diameter of the circular sensor, and capacitance results have been compared for the original shape and the transformed one, while taking into account the shape factor as in (8). The error due to geometry change is less than 1.5% except for the cases of small diameter and large thickness, as shown in Figure 9d. For the selected sensor properties and dimensions, the capacitance of the circular sensor was found to be 45.69pF, which is very close to 45.86pF of the square grid with a relative error of 0.36%. In conclusion, the selection of the ratio of perimeters to adjust the effects due to the change in shape is fully justified for the design and validation of SSFC of arbitrary shapes.

### 6.4. Characterization Output

The sensor characterization method results in several output data sets for the user-defined sweeps over materials, designs, and sizes as well as over force and thickness ranges. Such data sets can be used to analyze the transduction behavior of the SSFCs. These data sets also include the $nD\times nSS\times nS_{D}\times nM\times nT$ calibration curves. For instance, in the case of an SSFC that uses capacitive sensing, three calibration curves are shown in Figure 9e that describe the relationship between the applied force and the change in capacitance. These plots are fitted using linear regression and may be calibrated for field usage to determine the applied force from measured capacitance changes. Along with these plots, information related to dependence on sensing material properties and dimensions may be derived. For instance, Figure 9f shows that a sensing material of stiffness Y=1MPa with the dimensions chosen is not suitable for force sensing using capacitive transduction. Similarly, the plot in Figure 9g can help in selecting the appropriate thickness of the material for a given specification on sensor sensitivity. Furthermore, the transduction behavior profiles for different designs, sizes, and materials can be used to inform the selection of a high-accuracy yet ergonomic shoe design.

## 7. Conclusions

This paper has addressed the problem of accurate body weight estimation during walk using a novel shoe-integrated sensor architecture. The main concept is to concentrate the applied force using multi-layered, cleat-like structures that are called sandwiched sensor force consolidators (SSFC). Such structures are crucial in the reduction of the spreading-out effect of the ground-reaction forces. The paper then addressed the efficient characterization of such force consolidators using an equivalent-area methodology that can accurately account for SSFC’s with arbitrary shapes. In particular, a direct mapping has been derived that relates the sensed force to the SSFC dimensions and material properties. The issue of the computational efficiency of the equivalent-area characterization method has also been tackled and an OpenMP parallel programming interface has been implemented and shown to reduce the total processing time of a full shoe-integrated sensor array from few days to few hours. The design methodology has been implemented in a working prototype that has been fully tested using both static weights and the walking subjects. The achievable relative accuracy of body weight estimation was shown to be greater than 99%, *even* in the presence of motion. The achieved relative accuracy is at least 4X better than the existing state of the art.

While the major objective of the present work is the development of ergonomic, shoe-integrated sensors for medical body weight measurement, it is conceivable that the accuracy attained will enable more precise methods for several adjacent applications such as gait analysis, sports medicine, and physical therapy.

## Figures and Tables

**Table 1:** Medical Conditions Associated with Weight Changes.

Cancer	HIV/AIDS	Congestive Heart Failure	Fluid Retention (Oedema)
Diabetes, type 1/2	Anemia	Crohn’s disease	Tuberculosis
Hyperthyroidism	Chronic kidney disease	Peptic ulcer	Emphysema
Hypothyroidism	Ulcerative colitis	Polycystic ovary syndrome	Parkinson’s disease
Cystic fibrosis	Interstitial lung disease	Metabolic syndrome	Bowel disease
Thalassemia	Celiac disease	Lung cancer (non small cell)	Hypopituitarism
Multiple sclerosis	Malnutrition	Aortic aneurysm (abdomen)	Endocarditis
Helicobacter pylori infection	Addison’s Disease	Chronic obstructive pulmonary	Muscle loss
Sarcoidosis	Coeliac disease	Enzyme deficiencies	Anorexia
Hypercalcemia	Dementia	Cushing’s syndrome	Depression
Bulimia	Grief reaction	Excessive stress	

**Table 2:** Summary of Prior Art.

Ref Number	Sensor Used	Sensing Mechanism	Insole/External Shoe Frame	GRF Collection	Application	BWM ^a^	CSD ^b^	Technology Readiness
[5]	FSR array	Piezoresistive	Insole	Total	Biomechanics Lab	No	No	Commercial
[7]	FSR+LEDs	Piezoresistive+Optical	External	Total	Gait Analysis	No	No	Lab Prototype
[8]	FSR	Piezoresistive	Insole	Partial	Lifestyle Weight Monitoring	Yes	No	Lab Prototype
[9]	FSR	Piezoresistive	Insole	Partial	Estimation of Carried Load	Yes	No	Lab Prototype
[11]	FSR	Piezoresistive	Insole	Partial	Gait Recognition	No	No	Research
[12]	FSR	Piezoresistive	Insole	Partial	Gait Monitoring	No	No	Research
[13]	FSR	Piezoresistive	Insole	Partial	Vertical GRF Estimation	No	No	Research
[14]	FSR	Piezoresistive	Insole	Partial	Physical Therapy	No	No	Research
[15]	FSR	Piezoresistive	Insole	Partial	Stance Phase Recognition	No	No	Research
[16]	FSR	Piezoresistive	Insole	Partial	Low-cost GRF and CoP	No	No	Research
[17]	FSR	Piezoresistive	Insole	Partial	Gait Analysis	No	No	Research
[18]	FSR	Piezoresistive	Insole	Partial	Motion Analysis	No	No	Research
[19]	Pressure Sensors ^c^	Probbaly Piezoresistive	Insole	Partial	Real-time Gait Analysis	No	No	Licensed
[20]	Pressure Sensors ^c^	Probbaly Piezoresistive	Insole	Partial	Running Coach	No	Yes	Commercial
[21]	Pressure Sensors ^c^	Probbaly Piezoresistive	Socks	Partial	Personal Trainer	No	Yes	Commercial
[22]	Pressure Sensors ^c^	Probbaly Piezoresistive	Insole	Partial	Health Analytics	No	No	Commercial
[23]	Pressure Sensors ^c^	Probbaly Piezoresistive	Insole	Partial	Healthcare /Sports	No	Yes	Commercial
[24]	3-D tactile sensor array	Piezoresistive	External	Total	Tria-axial GRF	No	Yes	Research
[25]	Air pressure sensors	Air pressure	Insole ^d^	Total	Gait Monitoring	No	No	Lab Prototype
[26]	Air pressure sensors	Air pressure	Insole ^d^	Total	Activity Recognition	No	No	Lab Prototype
[27]	Fiber-optic force sensor	Intensity of light	External	Total	Biomechnaical Measurement	No	No	Research
[28]	Triaxial force sensors	Probably Piezoelectric	External	Total	Gait Variability Measurement	No	No	Research
[32]	Foam Sensors	Piezoelectric	Insole	Total	GRF during Walking	Yes	Yes	Lab Prototype
[29]	FSR	Piezoresistive	Insole	Total	GRF and Center of Pressure	No	No	Research
[30]	Optical Sensors	Intensity of light	Insole	Total	GRF during Jumping/Running	No	No	Research
[31]	Plantar Pressure (as in [5])	Probably Piezoresistive	Insole	Total	3D GRF and Frictional Torque	No	No	Lab Prototype
This Work	FSR	Piezoresistive	External	Total	Medical Weight Monitoring	Yes	Yes	Lab Prototype

^a^ Body Weight Measurement, ^b^ Custom Sensor Design, ^c^ Not Disclosed, ^d^ Insole consolidated sensors.

**Table 3:** Simulation Supported Physical Properties.

Transduction Mechanisms	Physical Property	Equation	Symbols
Capacitive	Capacitance	$C_{i,j}=\frac{\epsilon_{0}\epsilon_{r}lw}{\left(d-\Delta d_{i,j}\right)}$	$\epsilon_{0}$: Permittivity of free space $\epsilon_{r}$: Relative dielectric constant
Piezoelectric	Current	$I_{i,j}=\frac{e_{33}2\pi fF_{i,j}}{Y}$	$e_{33}$: Piezoelectric constant in the direction of force *f*: Force Frequency, *Y*: Young’s Modulus
Piezoresistive	Resistance	$R_{i,j}=\frac{(d-\Delta d_{i,j})\rho }{lw}$	ρ: Resistivity

## Abbreviations

The following abbreviations are used in this manuscript:

ADC	Analog to Digital Converter
BB	Bounding Box
FEM	Finite-Element-Method
FD	Force Distribution
FSR	Force-Sensing Resistor
GPU	Graphics Processing Unit
GRF	Ground Reaction Force
HPC	High-Performance Computing
OpenMP	Open Multi-Processing
SIM	Simulation
SSFC	Sandwiched Sensor Force Consolidators
ST	Shape Transformation
UART	Universal Asynchronous Receiver/Transmitter
US	United States
